# Immune Infiltration, Effector T-Cell Enrichment, and Functional Context for Prediction of Pathologic Complete Response to Neoadjuvant Chemotherapy in Breast Cancer

**DOI:** 10.3390/ijms27052431

**Published:** 2026-03-06

**Authors:** Ana Demšar, Klara Geršak, Barbara Gazić, Tanja Blagus, Katja Goričar, Gregor Jezernik, Vita Dolžan, Cvetka Grašič Kuhar

**Affiliations:** 1 Faculty of Medicine, University of Maribor, Taborska Ulica 8, 2000 Maribor, Slovenia; gregor.jezernik1@um.si; 2 Division of Medical Oncology, University Clinical Centre Maribor, Ljubljanska 5, 2000 Maribor, Slovenia; 3 General Hospital Izola, Polje 40, 6310 Izola, Slovenia; klara.gersak@sb-izola.si; 4 Faculty of Medicine, University of Ljubljana, Vrazov Trg 2, 1000 Ljubljana, Slovenia; bgazic@onko-i.si (B.G.); tanja.blagus@mf.uni-lj.si (T.B.); katja.goricar@mf.uni-lj.si (K.G.); vita.dolzan@mf.uni-lj.si (V.D.); cgrasic@onko-i.si (C.G.K.); 5 Division of Medical Oncology, Institute of Oncology Ljubljana, Zaloška Cesta 2, 1000 Ljubljana, Slovenia; 6 Department of Pathology, Institute of Oncology Ljubljana, Zaloška Cesta 2, 1000 Ljubljana, Slovenia

**Keywords:** breast cancer, neoadjuvant chemotherapy, pathological complete response, tumor-infiltrating lymphocytes, CD8-positive T cells, PD-1 immune checkpoint, immune microenvironment, predictive biomarkers

## Abstract

Tumor-infiltrating lymphocytes (TILs) are an established predictor of pathological complete response (pCR) after neoadjuvant chemotherapy (NACT) in breast cancer. However, TILs primarily reflect the extent of immune infiltration and provide limited insight into immune functional state. We investigated whether integrating measures of immune infiltration (TILs), effector T-cell presence (CD8), and functional context (immune checkpoint components) may improve prediction of pCR beyond TILs alone. Pretreatment tumor biopsies from 166 patients with early breast cancer treated with standard NACT were assessed for stromal TILs and mRNA expression of CD8, PD-1, LAG-3, and TIM-3. Associations with pCR were evaluated using univariate and multivariable logistic regression, and composite immune phenotypes were constructed to capture functional immune states. In univariate analyses, higher TILs, CD8, PD-1, and LAG-3 were associated with pCR (all *p* < 0.05), whereas TIM-3 was not (*p* = 0.801). In multivariable models, TILs remained independently associated with pCR when adjusted for checkpoint markers, but this association was attenuated when CD8 was included, consistent with the strong biological correlation between TILs and CD8, and neither CD8 nor checkpoint markers retained independent significance. PD-1 and LAG-3 expression strongly correlated with CD8 and moderately correlated with TILs, indicating that checkpoint expression predominantly reflects an immune effector–engaged tumor microenvironment. Composite immune phenotypes based on CD8/PD-1 co-expression identified distinct immune functional states, with CD8-high/PD-1-high tumors demonstrating the highest pCR rates. Hierarchical modeling showed modest improvements in discrimination with sequential addition of immune variables to clinical predictors, with the integrative CD8/PD-1 model achieving the highest discrimination within the cohort (AUC = 0.849), although the magnitude of improvement beyond TIL assessment alone was limited. In conclusion, immune infiltration, effector T-cell presence, and functional immune context represent complementary dimensions for pCR prediction following NACT in breast cancer. However, TILs remain the most robust and clinically feasible immune biomarker.

## 1. Introduction

Neoadjuvant chemotherapy (NACT) is a standard treatment approach for patients with high-risk early breast cancer, allowing tumor downstaging and providing an in vivo assessment of treatment response [1,2,3,4]. Achieving a pathological complete response (pCR) after NACT is strongly associated with improved long-term outcomes, particularly in HER2-positive and triple-negative breast cancer subtypes [5,6,7]. However, pCR rates vary widely among patients, underscoring the need for robust predictive biomarkers that can improve patient stratification and guide treatment decision-making.

Tumor-infiltrating lymphocytes (TILs) have emerged as one of the most reproducible immune-related biomarkers in breast cancer and have been consistently associated with higher pCR rates, particularly in triple-negative and HER2-positive disease [8,9,10]. Nevertheless, TILs primarily reflect the quantity of immune infiltration rather than the functional state of antitumor immunity, and their predictive performance remains heterogeneous across patients and tumor subtypes [10,11,12].

Cytotoxic CD8-positive T cells represent a key TIL effector population mediating antitumor immune responses. Increased CD8 infiltration has been associated with improved response to chemotherapy and favorable outcomes in breast cancer [13,14,15,16]. However, the presence of CD8-positive T cells alone does not necessarily indicate effective immune-mediated tumor control, as T-cell activity is tightly regulated by immune checkpoint pathways within the tumor microenvironment [17,18,19].

Programmed cell death protein 1 (PD-1) and other immune checkpoint receptors, including lymphocyte activation gene-3 (LAG-3) and T-cell immunoglobulin and mucin-domain containing-3 (TIM-3), are commonly expressed on tumor-infiltrating T cells. Their function is to mediate regulatory feedback that can attenuate T-cell function after sustained stimulation [17,18,19]. For this reason, these molecules are classically regarded as markers of immune inhibition or exhaustion; however, accumulating evidence suggests that in treatment-naïve tumors their expression may also reflect immune activation and ongoing antitumor immune engagement [15,20,21]. The biological and clinical significance of immune checkpoint expression in breast cancer remains incompletely understood, particularly in the setting of NACT in the absence of immune checkpoint blockade [22].

In this study, we aimed to evaluate the predictive value of baseline immune biomarkers for pCR in breast cancer patients treated with NACT. We assessed TILs, cytotoxic T-cell infiltration (CD8), and immune checkpoint expression (PD-1, LAG-3, and TIM-3) in pretreatment biopsies and hypothesized that these immune features are associated with treatment response and provide incremental predictive value beyond established clinical and pathological factors. By integrating measures of immune infiltration (TILs), effector T-cell presence (CD8), and functional context (immune checkpoint components), this work seeks to refine prediction of pCR and to explore the potential clinical utility of immune-informed risk stratification for neoadjuvant treatment de-escalation strategies.

## 2. Results

### 2.1. Patient Characteristics and Association with pCR

A total of 166 patients treated with NACT were included in the analysis. pCR was achieved in 55 patients (33.1%), while 111 patients (66.9%) did not achieve pCR (Table 1).

Patients who achieved pCR tended to be younger than those without pCR, although this difference did not reach statistical significance (mean age 46.4 ± 12.0 vs. 49.4 ± 10.6 years; *p* = 0.092). Tumor size was not significantly associated with pCR (median 26 mm vs. 27 mm; *p* = 0.142).

Clinical nodal status was significantly associated with treatment response (*p* = 0.009). Patients with node-positive disease were less likely to achieve pCR following NACT compared with node-negative patients (27.3% vs. 48.9%). In univariate logistic regression, nodal positivity was associated with a lower likelihood of pCR (OR 0.39, 95% CI 0.19–0.80; *p* = 0.010).

Tumor grade showed a strong association with pCR (*p* < 0.001). Grade 3 tumors were more likely to achieve pCR after NACT (40.7%) compared with grade 2 tumors (11.9%). Grade 3 disease was associated with increased odds of pCR (OR 5.07, 95% CI 1.86–13.79; *p* = 0.001).

Molecular subtype was significantly associated with pCR (*p* < 0.001). Compared with luminal B-like tumors, HER2-positive tumors had markedly higher odds of pCR following NACT (OR 7.04, 95% CI 3.12–15.88; *p* < 0.001), as did triple-negative tumors (OR 4.06, 95% CI 1.64–10.00; *p* = 0.002).

The proliferation marker Ki-67 was significantly higher in tumors achieving pCR (median 50% vs. 30%; *p* < 0.001). Increasing Ki-67 was associated with higher odds of pCR (OR 1.03 per 1% increase, 95% CI 1.01–1.05; *p* = 0.001).

### 2.2. Immune Biomarkers and pCR

Tumors achieving pCR after NACT exhibited significantly higher levels of immune infiltration and immune-related markers (Table 1). Median TIL levels were significantly higher in the pCR group compared with the non-pCR group (25% vs. 5%; *p* < 0.001), and increasing TIL percentage was associated with higher odds of pCR (OR 1.03 per 1% increase, 95% CI 1.01–1.04; *p* < 0.001).

**Figure 1:**
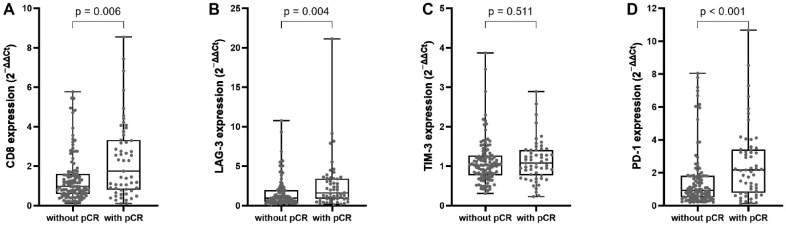
Immune marker expression according to pCR. Box-and-whisker plots compare normalized expression (2^−ΔΔCt^), normalized to the non-pCR group (set to 1), of (**A**) CD8, (**B**) LAG-3, (**C**) TIM-3, and (**D**) PD-1 between tumors achieving pCR and those without pCR. Higher expression of CD8 (*p* = 0.006), LAG-3 (*p* = 0.004), and PD-1 (*p* < 0.001) was observed in tumors with pCR, whereas TIM-3 showed no association with response (*p* = 0.511). Individual data points are shown; boxes represent the interquartile range with median, and whiskers indicate range. *p*-values were calculated using the Mann–Whitney U test.

CD8 expression was also significantly higher in tumors achieving pCR (median 1.75 vs. 0.97; *p* = 0.006, Figure 1) and was associated with increased likelihood of pCR in univariate analysis (OR 1.45, 95% CI 1.17–1.81; *p* < 0.001). Similarly, PD-1 expression was markedly higher in tumors achieving pCR (median 2.17 vs. 0.95; *p* < 0.001) and was associated with higher odds of pCR (OR 1.28, 95% CI 1.08–1.51; *p* = 0.004). LAG-3 expression was also higher in the pCR group (median 1.60 vs. 0.94; *p* = 0.004) and showed a modest association with pCR (OR 1.20, 95% CI 1.03–1.40; *p* = 0.023). In contrast, TIM-3 expression did not differ significantly between patients with or without pCR (*p* = 0.511) and was not associated with pCR in univariate analysis (OR 1.08, 95% CI 0.60–1.92; *p* = 0.801).

Correlation analysis (Supplementary Table S1) showed that TIL levels were moderately and positively associated with CD8 expression (r = 0.48, *p* < 0.001), indicating that higher immune infiltration is accompanied by increased cytotoxic T-cell presence. PD-1 and LAG-3 exhibited strong positive correlations with CD8 (r = 0.87, *p* < 0.001 and 0.76, *p* < 0.001, respectively) and moderate correlations with TILs (r = 0.46, *p* < 0.001 and 0.35, *p* < 0.001), indicating that immune checkpoint expression occurred predominantly in tumors characterized by immune infiltration and cytotoxic effector enrichment. In contrast, TIM-3 showed no meaningful correlation with TILs (r = 0.13) but moderate correlations with CD8, PD-1, and LAG-3 (r = 0.43–0.53, *p* < 0.001). Together, these patterns indicate that PD-1 and LAG-3 expression in this cohort primarily reflects an immune effector–infiltrated tumor microenvironment rather than intrinsic immune suppression.

To determine whether immune markers provided independent predictive value beyond immune infiltration and clinical factors, multivariable logistic regression models were constructed adjusting for nodal status, tumor subtype, proliferative index Ki-67, and TILs (Supplementary Table S2). In models incorporating immune checkpoint markers, TILs remained significantly associated with pCR after adjustment for clinical covariates (*p* = 0.024–0.045), whereas individual checkpoint markers, including PD-1 and LAG-3, were not statistically significant. In contrast, when CD8 expression was added to the TIL-based model, neither TILs nor CD8 remained independently significant. This attenuation across immune variables is consistent with substantial biological and statistical collinearity between overall immune infiltration, cytotoxic T-cell markers, and immune checkpoint expression.

### 2.3. Immune Phenotypes Based on CD8 and PD-1 Co-Expression and Prediction of pCR

To further explore the context-dependent role of immune checkpoint expression, tumors were stratified into four immune phenotypes based on combined CD8 and PD-1 expression, using median values to define low and high expression (Table 2). pCR rates differed significantly across these phenotypes (χ^2^ *p* = 0.003). Tumors with concurrent high CD8 and high PD-1 expression exhibited the highest pCR rate (46.4%), whereas tumors with high CD8 but low PD-1 expression showed the lowest pCR rate (6.7%). Intermediate pCR rates were observed in CD8-low/PD-1-high tumors and CD8-low/PD-1-low tumors. These findings indicate that CD8 infiltration alone is insufficient to optimally predict treatment response and that PD-1 expression gains predictive relevance specifically within a CD8-rich tumor microenvironment.

Additional immune checkpoint markers, including TIM-3 and LAG-3, were evaluated in combination with CD8 expression, but were not significantly associated with pCR and were therefore not included in subsequent predictive models (Supplementary Table S3).

### 2.4. Survival Outcomes According to Immune Phenotype

During a median follow-up of 5.3 years (range 0.1–7.4), 26 of 166 patients experienced recurrence, corresponding to an overall event rate of 15.7%. Event frequencies across immune phenotypes were 14/68 (20.6%) for CD8-low/PD-1-low tumors, 3/14 (21.4%) for CD8-low/PD-1-high tumors, 3/15 (20.0%) for CD8-high/PD-1-low tumors, and 6/69 (8.7%) for CD8-high/PD-1-high tumors. The estimated 5-year relapse-free survival (RFS) rates were 81.6%, 77.9%, 78.8%, and 91.2%, respectively (Supplementary Figure S1). Despite numerically favorable outcomes in the CD8-high/PD-1-high subgroup, survival differences between phenotypes were not statistically significant (log-rank χ^2^ = 4.16, *p* = 0.242).

### 2.5. Multivariable Logistic Regression Models Predicting pCR

**Figure 2:**
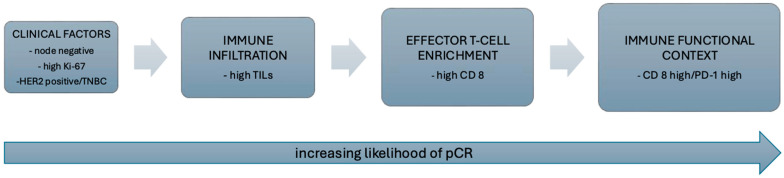
Hierarchical model of immune determinants of response to neoadjuvant chemotherapy. Schematic representation illustrating the hierarchical contribution of immune biomarkers to pathological complete response (pCR). Clinical factors reflect baseline tumor aggressiveness. Tumor-infiltrating lymphocytes (TILs) represent overall immune infiltration, while CD8 expression reflects cytotoxic effector T-cell enrichment. The CD8/PD-1 axis captures immune functional context, indicating immune engagement rather than terminal exhaustion. Integrating immune infiltration with effector T-cell enrichment and functional context was associated with the highest predictive performance in this cohort.

Hierarchically nested multivariable logistic regression models were constructed to assess the incremental predictive value of immune biomarkers beyond established clinical variables (Table 3). Models were specified sequentially to reflect biologically ordered immune dimensions, progressing from overall immune infiltration to cytotoxic effector enrichment and finally to immune functional context (Figure 2).

#### 2.5.1. Model 1: Clinical Model

The baseline clinical model included Ki-67, clinical nodal status, and molecular subtype. Higher Ki-67 was independently associated with increased odds of pCR (OR 1.05 per 1% increase, 95% CI 1.03–1.08, *p* < 0.001). Node-positive disease was associated with a significantly lower likelihood of pCR (OR 0.30, 95% CI 0.13–0.70, *p* = 0.005). Compared with luminal B tumors, HER2-positive tumors showed markedly increased odds of pCR (OR 14.52, 95% CI 5.35–39.41, *p* < 0.001), while triple-negative tumors were not independently associated with pCR in this model. The clinical model demonstrated moderate explanatory power (Nagelkerke R^2^ = 0.376).

#### 2.5.2. Model 2: Clinical Variables + TIL

Addition of TIL significantly improved model performance. Higher TIL levels were independently associated with increased odds of pCR (OR 1.03 per 1% increase, 95% CI 1.01–1.05, *p* = 0.003). Ki-67, nodal status, and HER2-positive subtype remained significant predictors. Model fit improved substantially (Nagelkerke R^2^ = 0.433).

#### 2.5.3. Model 3: Clinical Variables + TIL + CD8

To evaluate whether cytotoxic effector enrichment provided predictive value beyond overall immune infiltration, CD8 expression was added to the TIL-based model. In this model, CD8 showed a positive association with pCR that did not reach conventional statistical significance (OR 1.31, 95% CI 0.98–1.73, *p* = 0.065), while the effect of TILs was attenuated (OR 1.02, 95% CI 0.99–1.04, *p* = 0.089). Clinical covariates retained similar effect sizes. The concurrent attenuation of both TIL and CD8 effects in the combined model is consistent with substantial overlap between these variables, reflecting shared biological and statistical information between overall immune infiltration and cytotoxic effector enrichment. Inclusion of CD8 modestly improved model fit compared with the TIL-only model (Nagelkerke R^2^ = 0.453).

#### 2.5.4. Model 4: Clinical Variables + TILs + CD8 + CD8/PD-1 (Final Integrative Model)

The final model additionally incorporated the prespecified CD8-high/PD-1-high composite phenotype, contrasted against all other immune phenotypes, to assess whether this functionally defined cytotoxic immune state provided incremental predictive value beyond continuous CD8 expression. In the fully adjusted model, the composite demonstrated a positive but non-significant association with pCR (OR 1.55, 95% CI 0.45–5.35, *p* = 0.489). CD8 expression retained a positive but borderline association (OR 1.44, 95% CI 0.97–2.15, *p* = 0.074), while the effect of TILs was further attenuated (OR 1.02, 95% CI 1.00–1.04, *p* = 0.080). The concurrent attenuation of TIL, CD8, and the composite phenotype suggests substantial biological and statistical overlap among immune infiltration, effector enrichment, and functional context. Inclusion of the composite variable resulted in only a minimal improvement in model fit (Nagelkerke R^2^ = 0.456).

### 2.6. Model Discrimination and ROC Analysis

Model discrimination was assessed using receiver operating characteristic (ROC) analysis based on predicted probabilities derived from the hierarchically nested models (Table 4). The clinical model demonstrated good discrimination (AUC = 0.821). Addition of TILs improved discrimination (AUC = 0.844). Further inclusion of CD8 resulted in a modest additional increase (AUC = 0.847). Incorporation of the CD8-high/PD-1-high composite phenotype yielded only a minimal further increase in discrimination (AUC = 0.849). Consistent with the nested modeling framework, the largest gain in discrimination was observed with the inclusion of TILs, whereas subsequent additions of CD8 and immune functional context resulted in progressively smaller and clinically negligible improvements. ROC curves for all models are provided in the Supplementary Materials (Supplementary Figure S2).

### 2.7. Predictive Performance and Clinical Utility of the Integrative CD8/PD-1 Model

ROC analysis of the final integrative model (Clinical + TIL + CD8 + CD8/PD-1) demonstrated good discrimination for predicting pCR (AUC = 0.849). Using the optimal cutoff defined by the Youden index (predicted probability = 0.262), the model achieved a sensitivity of 90.9% and a specificity of 68.2% (Supplementary Table S4).

Given the clinical priority of minimizing undertreatment in the context of neoadjuvant therapy de-escalation, an alternative high-specificity threshold was evaluated. At a cutoff prioritizing specificity ≥ 95% (predicted probability = 0.694), the model achieved a specificity of 96.4% with a corresponding sensitivity of 32.7%. At this threshold, approximately 96% of patients who did not achieve pCR were correctly classified, limiting the theoretical risk of inappropriate de-escalation to approximately 4%, while identifying roughly one-third of patients who achieved pCR.

In this cohort, application of the high-specificity threshold would allow potential treatment de-escalation in 10.9% of patients using the integrative CD8/PD-1 model, compared with 10.8% using the Clinical + TIL model. This minimal absolute difference is consistent with the modest incremental improvement in discrimination observed across the hierarchically nested models.

## 3. Discussion

In this study, we investigated immunological predictors of pCR after NACT in early breast cancer. In addition to established predictive factors, including axillary lymph node status, molecular subtype, and the Ki-67 proliferation index, we confirmed the independent predictive value of TILs. Importantly, the composition of the TIL compartment appeared particularly relevant, as the combination of concurrently high CD8 and PD-1 expression demonstrated the strongest association with pCR among the evaluated immune markers. These findings suggest that refined immune phenotyping may improve identification of patients most likely to achieve pCR and potentially inform future strategies for NACT de-escalation.

Accurate prediction of pCR remains a major challenge and an area of ongoing need in the neoadjuvant treatment of breast cancer. While recent neoadjuvant trials have demonstrated incremental improvements in pCR rates through treatment escalation, such as the addition of immune checkpoint inhibitors in triple-negative and luminal B/HER2-negative disease, these strategies expose many patients to increased toxicity without guaranteed benefit [23,24,25]. Given the strong association between pCR and long-term outcomes, robust biomarkers capable of identifying patients likely to achieve pCR with standard therapy are critically needed to support safe de-escalation strategies [5,6,7].

### 3.1. Clinical Predictors of pCR

Consistent with prior studies, tumor subtype, nodal status, and proliferative activity (Ki-67) emerged as important clinical predictors of pCR in our cohort [6,7,8]. HER2-positive disease demonstrated the strongest association with pCR across all models, reflecting the established efficacy of HER2-targeted neoadjuvant therapy. Node-positive disease was consistently associated with lower odds of pCR, underscoring the adverse prognostic impact of nodal involvement. Ki-67 was a strong predictor of pCR in models incorporating only clinical variables or global immune infiltration, reflecting the greater chemosensitivity of highly proliferative tumors. In contrast, tumor size and patient age were not significantly associated with pCR. Large pooled analyses have shown that biological characteristics, particularly molecular subtype and immune context, outweigh tumor size as determinants of chemosensitivity [6,8]. Similarly, age has not been consistently shown to independently predict pCR, primarily influencing treatment tolerance rather than intrinsic tumor response [4,6].

### 3.2. Immune Infiltration and Treatment Response

The involvement of the immune system in response to NACT is well established, and TILs represent the most reproducible immune biomarker in breast cancer [8,9,10]. Our findings confirm prior reports demonstrating higher pCR rates in tumors with increased TILs, reinforcing their role as a marker of immune quantity and chemosensitivity. Mechanistically, several commonly used chemotherapeutic agents induce immunogenic cell death, characterized by the release of damage-associated molecular patterns which promote dendritic cell activation and priming of antitumor T-cell responses, thereby linking chemotherapy efficacy to a pre-existing immune-infiltrated tumor microenvironment [26,27].

### 3.3. Immune Effector Cell Component of TILs

Although stromal TILs provide a robust measure of immune infiltration, they represent a heterogeneous lymphoid population and offer limited insight into immune functionality [8,9]. Increasing evidence suggests that quantitative assessment of cytotoxic effector cells, particularly CD8^+^ T lymphocytes, may better capture the biologically relevant immune component driving treatment response [10,13,28]. This distinction is particularly relevant in luminal breast cancer, where baseline TIL levels are typically low and have limited predictive value for pCR compared with TNBC and HER2-positive disease [8,29], likely reflecting both sparse immune infiltration and a higher proportion of non-effector or bystander cells. Accordingly, intratumoral CD8^+^ density has been reported to discriminate treatment response more effectively than bulk TIL scoring in luminal B tumors, suggesting that effector T-cell quantification captures biologically relevant immune activity that is obscured by conventional TIL assessment [30].

In our cohort, both TILs and CD8 were significantly associated with pCR in univariate analyses; however, in multivariate models including both variables, neither retained statistical significance. This attenuation likely reflects substantial biological and statistical collinearity, given that CD8^+^ lymphocytes represent a major effector subset within the broader TIL compartment. When modeled together, their shared variance is partitioned between predictors, reducing apparent independent effects without diminishing their biological relevance. Consistent with prior evidence, these findings suggest that total immune infiltration and CD8^+^ effector enrichment capture overlapping but complementary dimensions of antitumor immunity. While TILs remain a practical and established biomarker, CD8-based measures may provide additional functional insight into immune activity.

### 3.4. Functional Context of Immune Checkpoint Expression

Higher PD-1 and LAG-3 expression were associated with increased pCR rates in univariate analyses, whereas TIM-3 was not. PD-1 and LAG-3 expression correlated strongly with TILs and CD8, indicating that it predominantly reflects immune-inflamed, antigen-engaged tumors rather than isolated immune suppression. These findings align with prior studies reporting that immune checkpoint expression in treatment-naïve breast cancer often reflects immune activation rather than terminal exhaustion, particularly in immune-inflamed tumors [15,31,32]. PD-1 expression has repeatedly been shown to correlate with increased TIL density and CD8^+^ T-cell infiltration and to associate with improved response to NACT in early breast cancer [15,33,34].

By contrast, the predictive relevance of LAG-3 and TIM-3 appears context dependent. While LAG-3 is enriched in immune-inflamed tumors and may associate with favorable outcomes in some cohorts, both LAG-3 and even more consistently TIM-3 have been linked to advanced T-cell exhaustion in chronic antigen exposure [19,35,36,37]. This heterogeneity suggests that these checkpoints may reflect later stages of immune dysfunction that are less responsive to chemotherapy-induced immune activation, explaining their limited predictive value in our analyses.

Our focus on immune checkpoint markers rather than additional cytotoxic effector molecules such as granzyme B or perforin was conceptually driven. Whereas cytotoxic mediators reflect active effector function, immune checkpoints provide insight into the regulatory and activation state of tumor-infiltrating T cells, including feedback inhibition or functional exhaustion. Evaluating this functional context was central to our objective of determining whether immune regulation, beyond effector presence alone, contributes to treatment response prediction.

### 3.5. Rationale for Composite Immune Biomarkers

From a statistical standpoint, the interdependence of TIL, cytotoxic effector and immune checkpoint markers introduces multicollinearity when correlated immune variables are modeled independently. Constructing biologically informed composite variables allows correlated markers to be integrated into a single immune phenotype, reducing redundancy while preserving meaningful information. Importantly, such composites encode immune states rather than additive effects. Only a limited number of prior studies have applied composite immune variables in breast cancer, but these demonstrate that combined assessment of immune infiltration and immune activation provides more informative prognostic and predictive insight than single markers alone [14,15,38,39,40,41].

Because PD-1 is upregulated upon T-cell activation, CD8 and PD-1 expression are biologically expected to co-occur, and in early treatment-naïve tumors, this pattern is increasingly interpreted as immune engagement rather than terminal exhaustion [15,20]. Modeling high CD8/PD-1 co-expression therefore captures a functionally activated immune microenvironment. Consistent with this concept, our results demonstrate that tumors with concurrent high CD8 and high PD-1 expression exhibited the highest rates of pCR, whereas CD8-high/PD-1-low tumors showed poor response, indicating that cytotoxic infiltration alone is insufficient in the absence of immune activation.

In exploratory survival analyses, the CD8-high/PD-1-high phenotype also demonstrated numerically favorable RFS, with the highest 5-year RFS rate among the four immune phenotypes. However, survival differences did not reach statistical significance, likely reflecting the limited number of recurrence events and restricted statistical power. These findings should therefore be interpreted as hypothesis-generating rather than definitive evidence of prognostic impact.

Together, these data indicate that in the neoadjuvant setting, high CD8/PD-1 co-expression reflects an activated immune microenvironment primed for chemotherapy-induced immunogenic cell death rather than immune exhaustion.

### 3.6. Immune Infiltration, Effector Presence, and Functional Context

Hierarchical incorporation of immune variables progressively improved model discrimination, from the clinical baseline to TILs, CD8, and ultimately the CD8/PD-1 composite, with the final integrative model achieving an AUC of 0.849 for prediction of pCR. Although this stepwise approach enhanced overall discrimination, the magnitude of improvement beyond TIL assessment alone was limited, and the CD8/PD-1 composite did not retain independent statistical significance, potentially reflecting both biological overlap and limited sample size. These findings support the concept that immune infiltration, effector cell presence, and functional immune context represent complementary dimensions of response prediction rather than competing biomarkers. However, while composite immune phenotypes may refine characterization of the tumor microenvironment, their added predictive value beyond TIL assessment appears modest in this cohort.

Prior studies have demonstrated that combinations of immune features outperform single markers in predicting pCR [14,15,38,39,40,41]. Notably, Goda et al. reported that a composite CD8/FOXP3 variable independently predicted response to neoadjuvant therapy after adjustment for tumor subtype and TILs, underscoring the clinical relevance of immune composites that integrate effector and regulatory compartments. Our findings align with this conceptual framework but indicate that the CD8/PD-1 composite, designed to reflect effector presence within a checkpoint-engaged context, provides only limited incremental predictive value in this cohort.

### 3.7. Clinical Translation and De-Escalation Potential

Predictive models intended to guide treatment de-escalation must prioritize high specificity to minimize undertreatment. At a specificity threshold ≥95%, approximately one third of patients in our cohort who achieved pCR could be correctly identified and considered for de-escalation. Incorporation of the CD8-high/PD-1-high composite into the TIL-based model did not meaningfully increase this proportion. Thus, under conservative clinical constraints, the studied immune biomarkers did not meaningfully improve identification of patients who could be safely spared overtreatment beyond TIL assessment.

Any incremental improvement in spared overtreatment must also be weighed against the technical, procedural, and financial complexity of mRNA-based immune profiling, which is not currently routine in clinical practice. mRNA-based quantification was selected to enable standardized, multiplex assessment of immune markers and objective comparison across samples within the analytical modeling framework. If the predictive signal associated with CD8/PD-1 composite variable was stronger and more robust, assessment by immunohistochemistry which is already widely implemented for both markers, could represent a more clinically feasible alternative. At present, however, TILs remain the most practical and readily applicable immune biomarker for pCR prediction in routine clinical settings.

### 3.8. Limitations

This study has several limitations. The sample size was modest, and the analysis was not powered for breast cancer subtype-specific modeling. Molecular subtype was included as a covariate rather than analyzed in stratified models in order to preserve statistical power; therefore, subtype-specific findings should be interpreted cautiously. The limited number of pCR events relative to the number of covariates represents a potential source of overfitting, particularly in the final integrative model. Although a hierarchical modeling strategy was employed to limit model complexity, these findings should be interpreted cautiously and validated in larger, independent cohorts.

Immune markers were assessed on biopsy specimens separate from those used for routine histopathological evaluation, potentially introducing spatial heterogeneity. In addition, immune biomarkers were measured at a single pretreatment time point, despite the dynamic nature of antitumor immunity. While CD8 and PD-1 expression were integrated to reflect immune functional activation within a cytotoxic T-cell-rich context, this composite phenotype represents a simplified approximation of immune states and does not capture additional regulatory or suppressive immune processes. Taken together, these limitations underscore the need for validation in larger, independent cohorts.

### 3.9. Conclusions and Future Directions

In conclusion, our study confirms the central role of the immune microenvironment in determining response to NACT in breast cancer. While TILs remain the most robust and clinically feasible immune biomarkers for pCR prediction, deeper immune characterization suggests that immune activation and functional context may refine predictive modeling, albeit with only modest incremental gain beyond TIL assessment alone. Given the complexity, spatial heterogeneity, and dynamic behavior of the antitumor immunity, single time-point biomarkers are unlikely to fully capture the biological determinants of treatment response. Future investigations incorporating larger, independent cohorts, longitudinal sampling, and integrative computational approaches, supported by emerging platforms for systematic tumor microenvironment profiling [42], will be necessary to validate and extend these findings and to enable safer and broader implementation of treatment de-escalation strategies.

## 4. Materials and Methods

### 4.1. Study Design and Patient Cohort

This prospective translational study was conducted within the framework of the AKRA study (Association of Immune Stromal Factors with Pathological Complete Response in Early Breast Cancer after Neoadjuvant Chemotherapy), performed at the Institute of Oncology Ljubljana between 2018 and 2021.

Eligible patients were ≥18 years old with histologically confirmed invasive breast cancer of luminal B-like, HER2-positive, or triple-negative subtype, clinical stage IIA (cT2N0M0) to IIIB (cT4a–cN2M0), and suitable for neoadjuvant systemic therapy. Patients with distant metastatic disease at diagnosis and patients with luminal-A-like tumors were excluded.

All patients received NACT according to contemporary European Society for Medical Oncology guidelines, consisting of anthracycline- and taxane-based regimens administered sequentially, with the addition of dual anti-HER2 therapy in HER2-positive disease [1]. Definitive breast surgery was performed 3–6 weeks after completion of neoadjuvant treatment.

pCR was defined as the absence of residual invasive carcinoma in the breast and axillary lymph nodes, allowing for the presence of carcinoma in situ (ypT0/Tis ypN0).

The study was conducted in accordance with the Declaration of Helsinki and approved by the Slovenian National Medical Ethics Committee (No. 0120-133/2017-2, dated 8 June 2017). All patients provided written informed consent prior to study inclusion.

### 4.2. Tumor Sampling and RNA Preservation

Pretreatment tumor tissue was obtained by ultrasound-guided core needle biopsy prior to the initiation of systemic therapy. During a single procedure, three to four cores were collected from each tumor; one core was designated for molecular analyses, while the remaining samples were used for routine histopathological evaluation. Specimens intended for molecular analyses were immediately immersed in an RNA-stabilizing solution (RNAlater Stabilization Solution, ThermoFisher, Waltham, MA, USA) and stored at −80 °C until RNA extraction.

### 4.3. Quantitative mRNA Expression Analysis

Immune-related biomarkers were assessed at the mRNA level by quantifying gene expression corresponding to key immune cell populations and immune checkpoint molecules. The analyzed genes included: *CD8A* (cytotoxic T-cell marker), *PDCD1* (PD-1), *LAG3* (LAG-3), *HAVCR2* (TIM-3).

#### 4.3.1. RNA Extraction from Biopsy Samples

Frozen pretreatment tumor biopsy samples were weighed prior to extraction, and up to 20 mg of tissue was used. Extraction was performed using the RNeasy Mini Kit (Qiagen, Hilden, Germany) with on-column DNase treatment (Qiagen) according to the manufacturer’s instructions. 2 M dithiothreitol (DTT) was used as the reducing agent. RNA concentrations were determined using the Lambda BIO+ UV/VIS (PerkinElmer, Waltham, MA, USA). RNA samples were stored at −80 °C.

#### 4.3.2. Reverse Transcription

To evaluate the expression of target genes, a total of 10 µL of total RNA, diluted to a maximum concentration of 60 ng/µL, was reverse transcribed using the High-Capacity cDNA Reverse Transcription Kit (Applied Biosystems, Waltham, MA, USA) according to the manufacturer’s instructions. The cDNA samples were stored at −20 °C until further analysis.

#### 4.3.3. Relative Expression Analysis

Detection and quantification of genes of interest and two endogenous controls (ACTB and CCSER2) were performed using the pre-defined TaqMan Gene Expression Assay (*CD8A* Hs00233520_m1, *HAVCR2* Hs00958618_m1, *PDCD1* Hs01550088_m1, *LAG3* Hs00958444_g1, *ACTB* Hs01060665_g1, *CCSER2* Hs00982799_mH, Applied Biosystems) and the TaqMan Fast Advanced Master Mix (Applied Biosystems, Waltham, MA. USA). All reactions were performed in technical triplicates and run on the QuantStudio 7 Flex Real-Time PCR System (Applied Biosystems) under the following thermal cycling conditions: 20 s at 95 °C, followed by 40 cycles of 1 s at 95 °C and 20 s at 60 °C. The analysis was performed using QuantStudio Software v1.7.2 (Applied Biosystems). Relative gene expression was calculated using the comparative Ct (2^−ΔΔCt^) method. A technical calibrator sample was included across runs to normalize between plates and minimize inter-run variability. For biological normalization, the mean ΔCt value of the non-pCR group was used as the internal calibrator for ΔΔCt calculations. Expression values therefore represent fold change relative to the non-pCR group, which was set to 1. These normalized values were used for subsequent statistical analyses.

### 4.4. Tumor-Infiltrating Lymphocyte (TIL) Assessment

Tumor-infiltrating lymphocytes were evaluated on hematoxylin and eosin-stained pretreatment biopsy sections according to the recommendations of the International TILs Working Group [9]. Stromal TILs were quantified as the percentage of stromal area occupied by mononuclear inflammatory cells and analyzed as a continuous variable. Representative examples of low and high stromal TIL levels are provided in Supplementary Figure S3.

### 4.5. Construction of Immune Composite Variables

To capture biologically meaningful immune context while reducing potential multicollinearity among closely related immune markers in multivariable models, composite immune variables were constructed. Selection of PD-1 for composite modeling was biologically prespecified rather than data-driven, as PD-1 expression is widely recognized as a marker of antigen-engaged T-cell activation. CD8 and PD-1 expression levels were dichotomized into high and low categories using cohort-specific median values. Based on these dichotomized variables, a composite CD8/PD-1 co-expression variable was generated, representing combined cytotoxic T-cell presence and immune checkpoint engagement. This approach was chosen to reflect immune functional state rather than isolated marker abundance. Four immune phenotypes were constructed based on combined CD8 and PD-1 status: CD8-low/PD-1-low, CD8-low/PD-1-high, CD8-high/PD-1-low, and CD8-high/PD-1-high. Associations between immune phenotypes and pCR were evaluated using the chi-square test, and their association with relapse-free survival was assessed using time-to-event analyses. Immune co-expression variables were evaluated primarily for their contribution to model performance rather than for precise estimation of individual effect sizes (small sample sizes within individual phenotype group).

Additional checkpoint markers (LAG-3 and TIM-3) were explored in combination with CD8 as exploratory composite variables and assessed for association with pCR using chi-square tests.

#### Survival Analysis

Relapse-free survival (RFS) was defined as the time from surgery to the first documented disease recurrence or death from any cause, whichever occurred first. Survival analyses were performed according to the four predefined immune phenotypes based on combined CD8 and PD-1 expression (CD8-low/PD-1-low, CD8-low/PD-1-high, CD8-high/PD-1-low, and CD8-high/PD-1-high). Patients without an event were censored at the date of last clinical contact. Survival analyses were prespecified exploratory endpoints. Survival distributions were estimated using the Kaplan–Meier method and compared between groups using the log-rank test. Median follow-up time was calculated using the reverse Kaplan–Meier method. Due to the limited number of events relative to candidate predictors, multivariable Cox regression analyses were not performed to avoid model overfitting.

### 4.6. Statistical Analysis

Continuous variables were assessed for normality using the Shapiro–Wilk test. Group comparisons were performed using Student’s *t*-test or the Mann–Whitney U test for continuous variables and χ^2^ or Fisher’s exact test for categorical variables, as appropriate. Gene expression variables were entered into regression models as relative expression values (2^−ΔΔCt^).

Univariate logistic regression analyses were conducted to assess associations between clinicopathological variables, immune biomarkers, and pCR. Odds ratios (ORs) with 95% confidence intervals (CIs) were reported. Correlations between continuous variables were evaluated using Spearman’s rank correlation coefficient.

Multivariable logistic regression models were first used to examine whether individual immune markers (CD8 and immune checkpoints) remained associated with pCR after adjustment for established clinicopathologic factors and TILs. Given the limited sample size and the number of candidate predictors, only variables demonstrating a statistically significant association with pCR in univariate analysis were carried forward into these adjusted models.

Immune-related variables were then introduced sequentially in a biologically motivated, hierarchical manner to assess their added predictive contribution. TILs were incorporated first to represent overall immune infiltration, followed by CD8 expression to capture cytotoxic effector cell presence beyond immune quantity, and finally immune functional state was introduced using a prespecified composite CD8/PD-1 variable reflecting concurrent high CD8 and high PD-1 expression, selected based on its established role as a marker of antigen-experienced T-cell activation.

In the multivariable logistic regression analysis, the CD8/PD-1 composite was coded as a binary indicator (CD8-high/PD-1-high vs. all other phenotypes), allowing assessment of whether a functionally defined cytotoxic immune phenotype provided incremental predictive information beyond cytotoxic T-cell presence. CD8 expression was retained in the final model to ensure that the composite phenotype was evaluated independently of continuous CD8 levels, thereby isolating the contribution of the functional immune state rather than allowing the composite to act as a surrogate for cytotoxic enrichment alone.

TILs were retained in all downstream models to preserve the biologically ordered structure of the hierarchical framework and to formally assess whether effector enrichment and immune functional context conferred additional predictive value beyond overall immune infiltration.

The following hierarchically nested models were evaluated within the same patient cohort:

1. Clinical model: Clinical variables only;
2. Immune infiltration model: Clinical variables plus TILs;
3. Effector enrichment model: Clinical variables plus TILs and CD8;
4. Immune functional context model: Clinical variables plus TILs, CD8 and CD8- high/PD-1-high composite variable.

Because these models were prespecified and hierarchically nested within an identical dataset, contribution of each immune layer was formally assessed using changes in −2 log likelihood. Changes in Nagelkerke R^2^ and area under the receiver operating characteristic curve (AUC) were additionally reported to describe shifts in explained variance and discrimination across hierarchically nested models following the sequential introduction of immune dimensions.

Receiver operating characteristic (ROC) analyses were performed using predicted probabilities from multivariable models. Optimal cutoffs were identified using the Youden index. In addition, high-specificity thresholds (≥95%) were evaluated to explore conservative treatment de-escalation strategies. Sensitivity, specificity, and proportions of patients potentially eligible for de-escalation were calculated descriptively.

All statistical analyses were performed using IBM SPSS Statistics (version 31.0). A two-sided *p* value < 0.05 was considered statistically significant.

### 4.7. Use of Artificial Intelligence Tools

During the preparation of this manuscript, generative artificial intelligence tools were used to assist with language refinement and structural editing. All outputs were reviewed, edited, and validated by the authors, who take full responsibility for the content of this publication.

## Figures and Tables

**Table 1:** Clinicopathological characteristics of patients according to pathological complete response (pCR) status.

Characteristics ^1^	All Patients ^2^ (n = 166)	Patients Without pCR (n = 111)	Patients with pCR ^3^ (n = 55)	*p*-Value ^4^ (Group Comparison)	OR (95% CI) ^5,6^	*p*-Value ^5^ (Univariate LR)
Age (years), mean ± SD	50.6 ± 11.2	49.4 ± 10.6	46.4 ± 12	0.092	0.97 (0.95–1.00)	0.094
Tumor size (mm), median (IQR)	26 (13)	27 (14)	26 (10)	0.142	0.98 (0.95–1.01)	0.104
Clinical nodal stage				0.009		
Node negative, n (%)	45 (27.1)	23 (51.1)	22 (48.9)		Ref.	
Node positive, n (%)	121 (72.9)	88 (72.7)	33 (27.3)		0.39 (0.19–0.80)	0.010
Grade				<0.001		
Grade 2, n (%)	42 (25.5)	37 (88.1)	5 (11.9)		Ref.	
Grade 3, n (%)	123 (74.5)	73 (59.3)	50 (40.7)		5.07 (1.86–13.79)	0.001
Molecular subtype				<0.001		
Luminal B like, n (%)	87 (52.4)	73 (83.9)	14 (16.1)		Ref.	
HER2-positive, n (%)	47 (28.3)	20 (42.6)	27 (57.4)		7.04 (3.12–15.88)	<0.001
Triple negative, n (%)	32 (19.3)	18 (56.2)	14 (43.8)		4.06 (1.64–10.00)	0.002
% Ki-67 median (IQR)	40 (28)	30 (30)	50 (30)	<0.001	1.03 (1.01–1.05)	0.001
% TIL median (IQR)	10 (25)	5 (19)	25 (45)	<0.001	1.03 (1.01–1.04)	<0.001
CD8 relative expression vs. non-pCR (2^−ΔΔCt^), median (IQR)	1.13 (1.95)	0.97 (1.03)	1.75 (2.53)	0.006	1.45 (1.17–1.81)	<0.001
LAG-3 relative expression vs. non-pCR (2^−ΔΔCt^), median (IQR)	1.10 (1.87)	0.94 (1.50)	1.60 (2.58)	0.004	1.20 (1.03–1.40)	0.023
TIM-3 relative expression vs. non-pCR (2^−ΔΔCt^), median (IQR)	1.04 (0.54)	1.03 (0.48)	1.08 (0.64)	0.511	1.08 (0.60–1.92)	0.801
PD-1 relative expression vs. non-pCR (2^−ΔΔCt^), median (IQR)	1.17 (1.72)	0.95 (1.31)	2.17 (2.63)	<0.001	1.28 (1.08–1.51)	0.004

^1^ Data are presented as mean ± SD (standard deviation), median (interquartile range (IQR)), or number (%), as appropriate. Gene expression values are presented as relative expression calculated using the 2^−ΔΔCt^ method, with the mean ΔCt value of the non-pCR group used as the internal biological calibrator (set to 1). Reported values are medians; therefore, the non-pCR group does not necessarily equal 1. ^2^ One value was missing for tumor grade and one for Ki-67; all other variables were complete. ^3^ Overall pCR rate was 33.1%. ^4^ Group comparisons were performed using Student’s *t*-test or Mann–Whitney U test for continuous variables and χ^2^ or Fisher’s exact test for categorical variables. ^5^ Odds ratios (ORs) and corresponding *p*-values were obtained from univariate logistic regression (LR) with pCR as the outcome. ^6^ Odds ratios for continuous variables represent change per one-unit increase.

**Table 2:** Immune phenotypes based on combined CD8 and PD-1 expression.

Immune Phenotype	Total (n)	No pCR n (%)	pCR n (%)
CD8-low/PD-1-low	68	52 (76.5)	16 (23.5)
CD8-low/PD-1-high	14	8 (57.1)	6 (42.9)
CD8-high/PD-1-low	15	14 (93.3)	1 (6.7)
CD8-high/PD-1-high	69	37 (53.6)	32 (46.4)
Total	166	111 (66.9)	55 (33.1)

Pearson chi-square *p* = 0.003.; percentages are shown per immune phenotype (row percentages). Associations between immune phenotypes and pCR were assessed using the chi-square test.

**Table 3:** Multivariable logistic regression models predicting pCR.

Variable	Model 1: Clinical n = 165 (pCR n = 55)	Model 2: Clinical + TIL n = 165 (pCR n = 55)	Model 3: Clinical + TIL + CD8 n = 165 (pCR n = 55)	Model 4: Clinical + TIL + CD8 + CD8/PD-1 n = 165 (pCR n = 55)
	OR (95% CI), *p*-Value	OR (95% CI), *p*-Value	OR (95% CI), *p*-Value	OR (95% CI), *p*-Value
**Ki-67**	1.05 (1.03–1.08), <0.001	1.05 (1.02–1.07), <0.001	1.05 (1.03–1.08), <0.001	1.05 (1.03–1.08), <0.001
**Node-positive**	0.30 (0.13–0.70), 0.005	0.22 (0.09–0.56), 0.001	0.21 (0.08–0.53), 0.001	0.20 (0.08–0.52), <0.001
**HER2-positive**	14.52 (5.35–39.41), <0.001	15.01 (5.22–43.12), <0.001	15.10 (5.14–44.38), <0.001	16.00 (5.37–47.73), <0.001
**Triple negative**	1.94 (0.69–5.46), 0.211	1.79 (0.62–5.17), 0.284	1.68 (0.57–4.97), 0.346	1.73 (0.58–5.14), 0.326
**TILs**	—	1.03 (1.01–1.05), 0.003	1.02 (0.99–1.04), 0.089	1.02 (1.00–1.04), 0.080
**CD8**	—	—	1.31 (0.98–1.73), 0.065	1.44 (0.97–2.15), 0.074
**CD8/PD-1**	—	—	—	1.55 (0.45–5.35), 0.489
**Nagelkerke R^2^**	0.376	0.433	0.453	0.456

pCR was coded as the outcome variable (1 = pCR, 0 = no pCR). Odds ratios (ORs) are reported with 95% confidence intervals. For continuous variables, ORs represent the change in odds per unit increase. Reference categories were luminal B subtype and node-negative disease. Hierarchically nested models were constructed sequentially to evaluate the incremental predictive contribution of immune variables beyond clinical factors. In the final model, the composite CD8/PD-1 variable was coded as a binary indicator contrasting CD8-high/PD-1-high tumors with all other phenotypes.

**Table 4:** Model performance and discrimination.

Model	−2 Log Likelihood	Nagelkerke R^2^	AUC
Model 1: Clinical	157.99	0.376	0.821
Model 2: Clinical + TIL	148.35	0.433	0.844
Model 3: Clinical + TIL + CD8	144.85	0.453	0.847
Model 4: Clinical + TIL + CD8 + CD8/PD-1	144.36	0.456	0.849

Model discrimination was assessed using the area under the receiver operating characteristic curve (AUC), calculated from predicted probabilities derived from each hierarchically nested logistic regression model. Higher AUC values indicate improved discrimination. Model fit was evaluated using −2 log likelihood (−2LL) and Nagelkerke R^2^. All models were fitted on complete cases within the same analytic cohort (n = 165), enabling direct comparison of performance metrics across nested models.

## Data Availability

The original contributions presented in this study are included in the article/Supplementary Materials. Further inquiries can be directed to the corresponding author.

## Supplementary Materials

The following supporting information can be downloaded at: https://www.mdpi.com/article/10.3390/ijms27052431/s1.

## Abbreviations

ADC	Antibody–drug conjugate
AUC	Area under the curve
CD8	Cluster of differentiation 8
H&E	Hematoxylin and eosin
HER2	Human epidermal growth factor receptor 2
IHC	Immunohistochemistry
LAG-3	Lymphocyte activation gene 3
mRNA	Messenger ribonucleic acid
NACT	Neoadjuvant chemotherapy
OR	Odds ratio
pCR	Pathologic complete response
PD-1	Programmed cell death protein 1
ROC	Receiver operating characteristic
RFS	Relapse-free survival
TIM-3	T-cell immunoglobulin and mucin-domain containing protein 3
TILs	Tumor-infiltrating lymphocytes
TNBC	Triple-negative breast cancer
