# Supplementary material for: Immune Infiltration, Effector T-Cell Enrichment, and Functional Context for Prediction of Pathologic Complete Response to Neoadjuvant Chemotherapy in Breast Cancer
Source: Int J Mol Sci. 2026 Mar 6;27(5):2431. doi: 10.3390/ijms27052431 (PMC12985902; doi:10.3390/ijms27052431)
Supplement: Supplementary file 1 [file ijms-27-02431-s001.zip › ijms-4157149-supplementary.pdf]

Supplementary Table S1. Correlations between proliferation, immune infiltration, and immune checkpoint markers
Spearman's rank correlation coefficients (ϱ)

Variable	Ki-67	TILs	CD8A	PD-1	LAG-3	TIM-3
**Ki-67**	1.00	0.29***	0.20*	0.20*	0.19*	0.04
**TILs**	0.29***	1.00	0.48***	0.46***	0.35***	0.13
**CD8**	0.20*	0.48***	1.00	0.87***	0.76***	0.43***
**PD-1**	0.20*	0.46***	0.87***	1.00	0.86***	0.53***
**LAG-3**	0.19*	0.35***	0.76***	0.86***	1.00	0.43***
**TIM-3**	0.04	0.13	0.43***	0.53***	0.43***	1.00

n = 165–166 (1 patient without Ki67 value)
* $p < 0.05$
** $p < 0.01$
*** $p < 0.001$

Supplementary Table S2. Multivariable logistic regression models for prediction of pathological complete response (pCR)

Clinical variables + TILs + CD8

Variable	OR (95% CI)	*p*-value
**Ki-67**	1.051 (1.025–1.079)	<0.001
**Node-positive**	0.208 (0.081–0.534)	0.001
**HER2+**	15.098 (5.136–44.384)	<0.001
**Triple-negative**	1.683 (0.570–4.967)	0.346
**TILs**	1.019 (0.997–1.040)	0.089
**CD8**	1.307 (0.983–1.737)	0.065

Model performance: Nagelkerke $R^2 = 0.453$

Clinical variables + TILs + PD-1

Variable	OR (95% CI)	p-value
**Ki-67**	1.047 (1.022–1.073)	<0.001
**Node-positive**	0.215 (0.084–0.534)	0.001
**HER2+**	15.522 (5.330–45.139)	<0.001
**Triple-negative**	1.754 (0.604–5.100)	0.302
**TILs**	1.022 (1.000–1.045)	0.045
**PD-1**	1.124 (0.883–1.431)	0.341

Model performance:Nagelkerke $R^2 = 0.439$

Clinical variables + TILs + LAG-3

Variable	OR (95% CI)	p-value
**Ki-67**	1.047 (1.021–1.079)	<0.001
**Node-positive**	0.220 (0.087–0.556)	0.001
**HER2+**	8.879 (2.503–31.500)	<0.001
**Triple-negative**	0.562 (0.193–1.634)	0.289
**TILs**	1.024 (1.003–1.047)	0.024
**LAG-3**	1.098 (0.883–1.366)	0.391

Model performance: Nagelkerke $R^2 = 0.438$

Multivariable logistic regression analyses evaluating clinical and immune predictors of pathological complete response (pCR). Odds ratios (ORs) are reported with 95% confidence intervals (CI). Continuous variables (Ki-67, TILs, CD8, and PD-1) are modeled per unit increase. Reference categories were luminal B subtype for molecular subtype and node-negative disease for nodal status. For all models n=165 (pCR n=55).

Supplementary Table S3. Immune phenotypes stratified by pathological complete response (pCR)

Immune phenotype	No pCR, n (%)	pCR, n (%)	Total (n)
CD8 Low / LAG3 Low	38 (58.8)	27 (41.2)	65
CD8 Low / LAG3 High	14 (73.7)	5 (26.3)	19
CD8 High / LAG3 Low	50 (56.9)	38 (43.1)	88
CD8 High / LAG3 High	9 (64.3)	5 (35.7)	14
**Total**	**111 (66.9)**	**55 (33.1)**	**166**

Pearson $\chi^2 = 6.76$, df = 3, $p = 0.080$.

Immune phenotype	No pCR, n (%)	pCR, n (%)	Total (n)
CD8 Low / TIM3 Low	38 (71.7)	15 (28.3)	53
CD8 Low / TIM3 High	22 (75.9)	7 (24.1)	29
CD8 High / TIM3 Low	21 (65.6)	11 (34.4)	32
CD8 High / TIM3 High	30 (57.7)	22 (42.3)	52
**Total**	**111 (66.9)**	**55 (33.1)**	**166**

Pearson $\chi^2 = 3.62$, df = 3, $p = 0.306$.

Percentages are shown per immune phenotype (row percentages). Associations between immune phenotypes and pathologic complete response were assessed using the chi-square test.

Supplementary Table S4. Diagnostic performance of the Clinical + TIL and Clinical + TIL + CD8 + CD8/PD-1 models at predefined thresholds

Model	Cutoff strategy	Cutoff	Sensitivity (%)	Specificity (%)	Correctly spared overtreatment (%)
Clinical + TIL	Youden index	0.29	90.9	70.9	—
Clinical + TIL	High specificity	0.72	32.7	96.0	10.8
Clinical + TIL + CD8 + CD8/PD-1	Youden index	0.26	90.9	68.2	—
Clinical + TIL + CD8 + CD8/PD-1	High specificity	0.63	32.7	96.4	10.9

Sensitivity and specificity were calculated using predicted probabilities derived from multivariable logistic regression models. The optimal cutoff was defined using the Youden index. A high-specificity threshold (≈ 96%) was additionally evaluated to reflect a conservative neoadjuvant treatment de-escalation strategy. "Correctly spared overtreatment" represents the proportion of the total cohort correctly identified as achieving pCR at the high-specificity threshold and who would hypothetically be considered for treatment de-escalation in a future, biomarker-guided setting. All analyses are exploratory and intended to illustrate potential clinical trade-offs rather than establish definitive decision thresholds.

Supplementary Figure S1. Relapse-free survival according to immune phenotype defined by combined CD8 and PD-1 expression

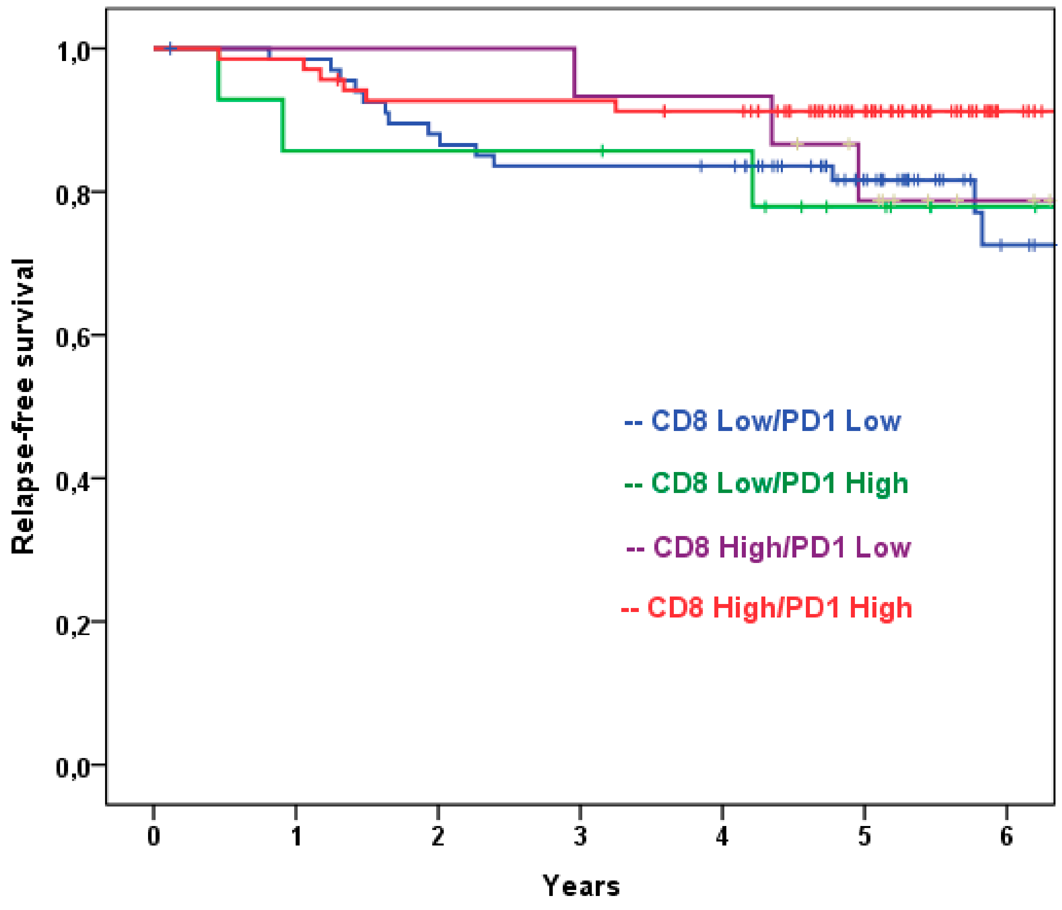

At risk

CD8 Low/PD1 Low	68	66	59	56	55	37	15
CD8 Low/PD1 High	14	12	12	12	11	7	3
CD8 High/PD1 Low	15	15	15	14	14	10	5
CD8 High/PD1 High	69	68	63	63	61	43	15

Legend: Kaplan–Meier curves for relapse-free survival stratified by immune phenotypes based on combined CD8 and PD-1 expression levels (median cutoffs): CD8-low/PD-1-low, CD8-low/PD-1-high, CD8-high/PD-1-low, and CD8-high/PD-1-high. Tick marks indicate censored observations. Differences between groups were not statistically significant (log-rank $\chi^2 = 4.16$, $p = 0.242$).

Supplementary Figure S2. Receiver Operating Characteristic (ROC) Curves for Prediction of Pathological Complete Response (pCR)

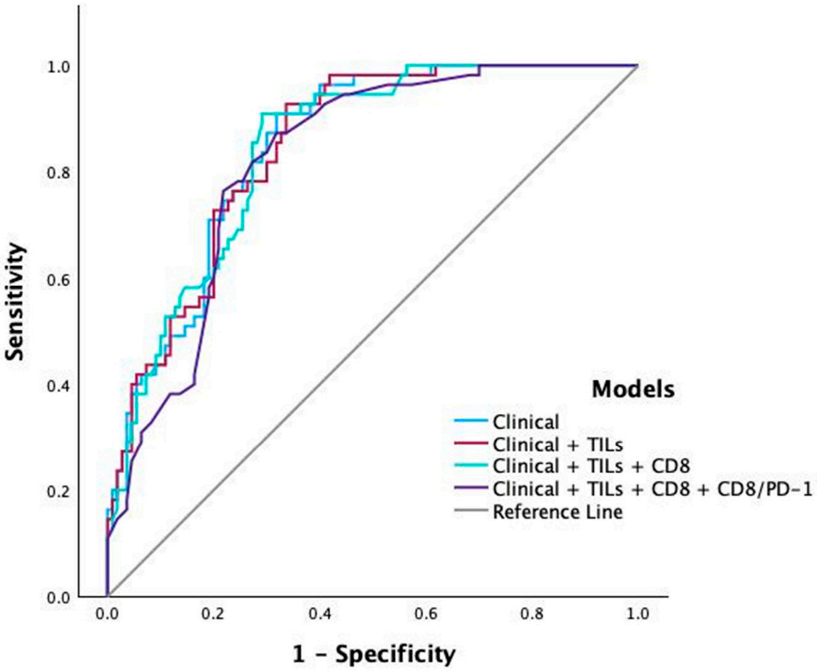

Comparison of predictive performance for sequentially constructed models:
Clinical variables alone; Clinical + TILs; Clinical + TILs + CD8; and the integrative Clinical + TILs + CD8 + CD8/PD-1 composite model. The reference line represents random classification.

Supplementary Figure S3. Representative H&E Images for Tumor-Infiltrating Lymphocyte (TIL) Assessment

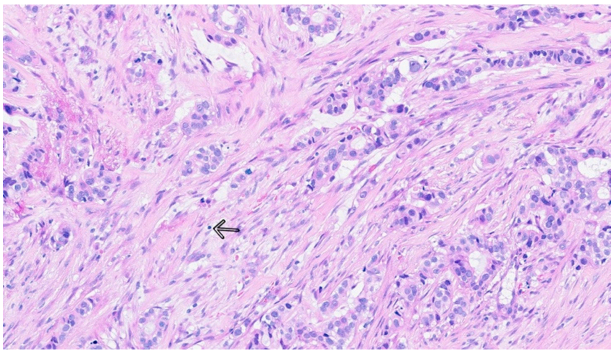

(A) Low stromal TIL density (2%) in pretreatment tumor specimen.

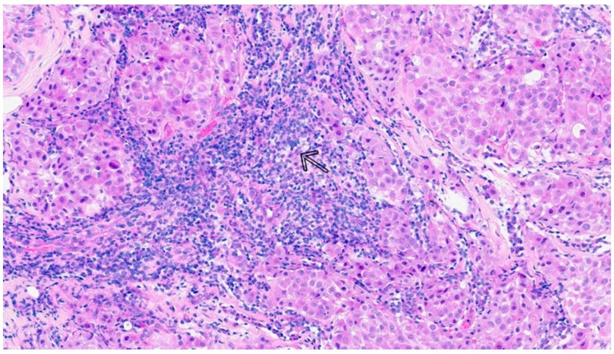

(B) High stromal TIL density (80%) in pretreatment tumor specimen.

Images are shown at 20× magnification. Arrows indicate representative stromal tumor-infiltrating lymphocytes identified according to standardized TIL assessment criteria.
